# Indocyanine Green-Loaded Quenched Nanoliposomes as Activatable Theranostics for Cancer

**DOI:** 10.3390/molecules30071414

**Published:** 2025-03-22

**Authors:** Junwoo Lim, Yeojin Yoo, Yongdoo Choi

**Affiliations:** Division of Technology Convergence, National Cancer Center, 323 Ilsan-ro, Goyang 10408, Gyeonggi-Do, Republic of Korea; jwlim@ncc.re.kr (J.L.); yooyeos@ncc.re.kr (Y.Y.)

**Keywords:** activatable, indocyanine green, nanoliposome, phototherapy

## Abstract

Photodynamic therapy (PDT) and photothermal therapy (PTT) are considered to be one of the most effective methods for treating cancer due to their noninvasive nature, high effectiveness, and fewer side effects compared to standard therapeutic modalities for cancer. However, conventional always-on types of PDT and PTT agents have basic drawbacks in their in vivo applications, which include the unwanted generation of strong fluorescence signals and phototoxicity in normal tissues, including blood vessels, when exposed to light, resulting in poor imaging contrast and unwanted phototoxicity. Here, we propose indocyanine green-loaded quenched nanoliposomes (Q-ICG-NLs) as an activatable nanotheranostics. Q-ICG-NLs showed significant quenching in near-infrared fluorescence emission and singlet oxygen generation upon light irradiation. The photothermal effect of Q-ICG-NLs was 1.3 times greater than free indocyanine green. Its fluorescence and singlet oxygen generation were largely restored when taken up into cancer cells, enabling the selective detection and phototherapy of cancer cells. These results suggest that Q-ICG-NLs can be effectively used for selective near-infrared fluorescence imaging and the subsequent image-guided PDT and PTT of cancers.

## 1. Introduction

Photodynamic therapy (PDT) and photothermal therapy (PTT) using chemical photosensitizers are among the most effective methods for treating cancer due to their noninvasive nature, high effectiveness, and fewer side effects compared to standard therapeutic modalities for cancer such as surgery, chemotherapy, and radiotherapy [1,2,3]. Near-infrared (NIR) light irradiation is particularly advantageous for PDT and PTT because it allows for relatively deep tissue penetration due to the low photon absorption by endogenous molecules within the NIR wavelength range [4,5]. Additionally, PDT and PTT can selectively kill cancer cells at the site of light irradiation while sparing normal tissues without exposure to light. However, “always-on” types of phototherapy agents, especially PDT agents, have inherent limitations in cancer fluorescence imaging and therapeutic efficacy. Specifically, when exposed to light, they emit strong fluorescence signals and generate reactive oxygen species, regardless of whether they are in cancerous or normal tissues, resulting in poor imaging contrast and unwanted phototoxic effect to normal cells [6]. These drawbacks significantly diminish the effectiveness of “always-on” PDT agents in in vivo applications. To address these limitations, extensive research has been conducted to develop advanced PDT agents and delivery systems, although this field is still evolving [7,8,9].

**Figure 1:**
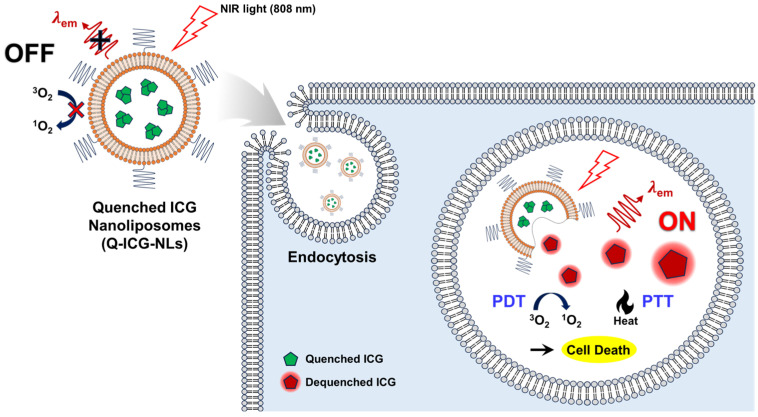
A schematic illustration of Q-ICG-NLs demonstrates their application in activatable NIR fluorescence imaging, photodynamic therapy (PDT), and photothermal therapy (PTT) for cancer treatment. In normal tissues or the extracellular environment, the near-infrared (NIR) fluorescence and singlet oxygen generation of ICG molecules are mostly quenched. However, after Q-ICG-NLs accumulate in tumors via the enhanced permeation and retention (EPR) effect and are internalized by cancer cells, ICG molecules are released from the nanoliposomes, restoring strong fluorescence and enhancing phototoxicity. Furthermore, the imaging-guided PTT induces heat stress can amplify phototoxicity to the cancer cells [17].

Indocyanine green (ICG), widely used as a photosensitizer and photothermal agent, is a NIR fluorescent dye that has been clinically applied in angiography, sentinel lymph node mapping, and intraoperative imaging [10,11]. ICG exhibits excitation and emission in the NIR wavelength range, enhancing tissue penetration depth and improving tumor eradication [12]. However, ICG rapidly binds to plasma proteins in vivo, leading to fast clearance from the bloodstream [13]. It also shows poor optical, thermal, and chemical stability in aqueous environments due to aggregation, limiting its development as a theranostic agent [14,15]. Additionally, ICG undergoes rapid photodegradation upon light exposure, resulting in low efficiency [5,15]. Its fluorescence is further compromised by molecular aggregation in aqueous conditions. Although nano-delivery systems have been developed to overcome these limitations, they still encounter challenges similar to those faced by “always-on” PDT agents [7,16,17]. In this study, we developed quenched ICG-loaded nanoliposomes (Q-ICG-NLs) as an activatable theranostic agent (Figure 1). The NIR fluorescence and singlet oxygen generation (SOG) of Q-ICG-NLs are significantly quenched when formulated as nanoliposomes. However, once Q-ICG-NLs enter cancer cells and release the encapsulated ICG molecules, both fluorescence and SOG are restored, allowing for tumor-selective fluorescence imaging and image-guided precision phototherapy. The photothermal effect of Q-ICG-NLs was also evaluated and compared with that of free ICG. These nanoliposome-based off–on agents, such as Q-ICG-NLs, are expected to be effective for selective fluorescence activation and phototherapeutic cancer treatment, thereby facilitating fluorescence image-guided precision PDT and PTT.

## 2. Results and Discussion

### 2.1. Preparation and Characterization of Q-ICG-NLs

To prepare the Q-ICG-NLs, a mixture of lipids and ICG was first coated onto a round-bottom flask. Distilled water was then added, followed by sonication to induce liposomal formation. The ICG-loaded nanoliposomes were subsequently purified using size exclusion chromatography and centrifugation. Nanoliposomes were prepared by varying the ratio between ICG and phospholipids, and the fluorescence intensities of the prepared nanoliposomes were then measured. As shown in Figure S1, the NIR fluorescence of the nanoliposomes was significantly quenched as the loading content of ICG molecules increased. The optimal ICG loading content for achieving the maximal quenching effect was determined to be 0.3 mg/mL (ICG:lipid molar ratio = 1:25), and this condition was used to prepare Q-ICG-NLs for subsequent experiments.

**Figure 2:**
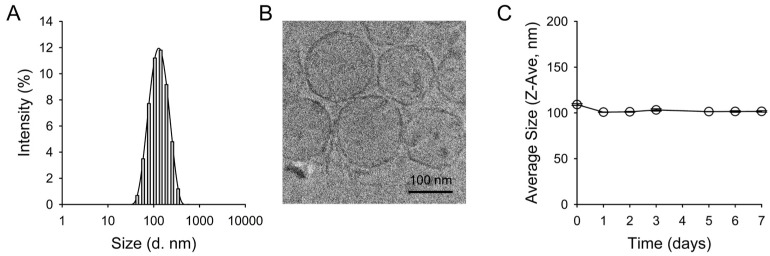
(**A**) Hydrodynamic size distribution of Q-ICG-NLs. (**B**) Cryo-TEM image of Q-ICG-NLs showing their round-shaped unilamellar structure. (**C**) Stability analysis of Q-ICG-NLs over a 7-day period by monitoring changes in the hydrodynamic size.

The hydrodynamic size of the prepared Q-ICG-NLs was 109.1 ± 1.0 nm (Figure 2A). Cryo-transmission electron microscopy (Cryo-TEM) imaging of Q-ICG-NLs revealed a round-shaped unilamellar structure (Figure 2B). The nanoliposome size measured using the cryo-TEM images was 107.9 ± 26.7 nm. The stability of Q-ICG-NLs was also evaluated, showing no significant changes in size distribution over a 7-day period (Figure 2C). The encapsulation efficiency of ICG in the nanoliposomes was 80.8 ± 5.3%.

**Figure 3:**
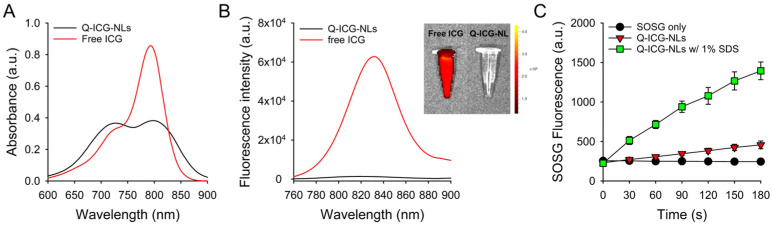
(**A**) Representative UV/Vis absorption and (**B**) fluorescence spectra of Q-ICG-NLs and free ICG in deionized water. Fluorescence images of microtubes containing free ICG and Q-ICG-NLs are shown in the inset of (**B**). (**C**) Measurement of singlet oxygen generation (SOG) using Q-ICG-NLs and Q-ICG-NLs containing 1% SDS during 808 nm laser irradiation (*n* = 4).

The UV/Vis absorption spectrum of Q-ICG-NLs in deionized water showed a significant increase in the relative intensity of the shoulder peak at 715 nm, while the peak of monomeric ICG at 792 nm decreased compared to that of free ICG. These data indicate the formation of H-aggregates of ICG molecules within the nanoliposomes (Figure 3A) [18]. Correspondingly, the NIR fluorescence of Q-ICG-NLs was effectively quenched (Figure 3B). In vitro fluorescence images further demonstrated that the NIR fluorescence of Q-ICG-NLs was almost completely suppressed compared to that of free ICG, confirming the efficient quenching of ICG within the nanoliposomes.

Subsequently, singlet oxygen generation (SOG) was evaluated using singlet oxygen sensor green (SOSG), a reagent for the selective detection of ^1^O_2_ production. Upon light irradiation, the fluorescence intensity of SOSG in the Q-ICG-NL sample slightly increased over time. However, when Q-ICG-NLs were mixed with a 1% sodium dodecyl sulfate (SDS) solution to disrupt the nanoliposomal formulation and release ICG molecules from the nanoliposomes, the SOSG fluorescence significantly increased over time, indicating a recovery of ^1^O_2_ generation capability. Changes in the UV/Vis absorption and fluorescence spectra of Q-ICG-NLs after addition of 1% SDS solution (Figure S2) also indicate that the disaggregation and release of ICG molecules from the nanoliposomes result in the dequenching of the optical properties of Q-ICG-NLs. These data confirm that the SOG of Q-ICG-NLs was effectively quenched, similar to its NIR fluorescence, and that the quenched SOG could be recovered when ICG molecules were released from the nanoliposomes.

### 2.2. Photothermal Effects of Q-ICG-NLs and ICG

**Figure 4:**
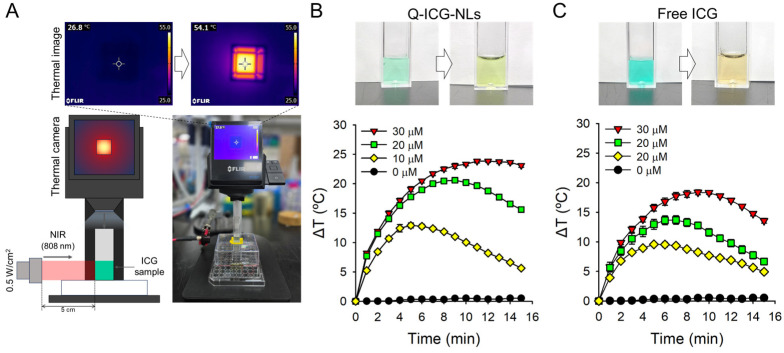
Observation of photothermal effects. (**A**) Schematic illustration of the experimental setup for photothermal effect analysis and temperature imaging, demonstrating a heat map within the disposable cuvette. Time-dependent temperature changes in (**B**) Q-ICG-NLs and (**C**) free ICG in aqueous solution during 808 nm laser irradiation. Photographs taken before and after light irradiation are shown in the upper images.

The time-dependent photothermal effect of Q-ICG-NLs and free ICG under NIR light irradiation was analyzed and compared. The thermal response of Q-ICG-NLs was significantly amplified upon exposure to 808 nm light. Sequential heatmap images depicting the progressive thermal effects on Q-ICG-NLs under light irradiation are shown in Figure 4A. Solutions containing both Q-ICG-NLs and free ICG exhibited a concentration-dependent increase in temperature upon light irradiation. Notably, Q-ICG-NLs showed a higher peak temperature compared to free ICG and maintained this elevated temperature for a longer duration. For instance, at an ICG-equivalent concentration of 30 µM, Q-ICG-NLs demonstrated a 1.3-fold greater peak temperature compared to free ICG, highlighting a substantial enhancement in the photothermal effect. In contrast, the temperature in the control sample containing phosphate-buffered saline (PBS; pH 7.4, 10 mM, 136 mM NaCl) increased by only 0.1 °C. Previous studies have shown that irreversible cellular damage occurs after 1 h of exposure to 46 °C, while exposure to temperatures of 50–52 °C for only 4–6 min can induce similar effects [19,20]. Additionally, the photothermal effect induced heat stress, which further triggered heat-induced reactive oxygen species generation, thereby enhancing phototoxicity in cancer cells [21]. These findings suggest that Q-ICG-NLs are highly effective in enhancing phototherapy against cancer cells.

### 2.3. Cellular Uptake of Q-ICG-NLs and the Subsequent Recovery of Their NIR Fluorescence

**Figure 5:**
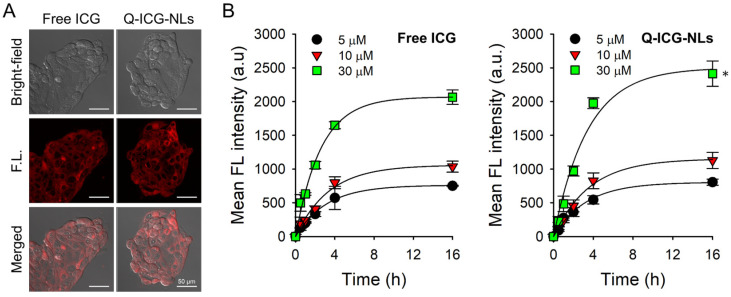
Evaluation of the fluorescence recovery of Q-ICG-NLs after intracellular uptake. (**A**) Confocal fluorescence images of Calu-3 cells treated with Q-ICG-NLs and free ICG (λ_ex._ = 633 nm, λ_em._ = 700 ± 50 nm). Scale bar = 50 μm. (**B**) Quantitative analysis of NIR fluorescence signals in Calu-3 cells treated with Q-ICG-NLs and free ICG (*n* = 4, * *p* < 0.05).

Next, we investigated the intracellular uptake and subsequent fluorescence recovery of Q-ICG-NLs using in vitro cell studies. Calu-3 human lung adenocarcinoma cells were treated with Q-ICG-NLs or free ICG at ICG-equivalent concentrations of 5, 10, and 30 µM for 16 h. After washing the cells, confocal imaging was performed (Figure 5A). Notably, cells exposed to Q-ICG-NLs and free ICG exhibited similarly strong fluorescence intensities at the tested concentrations. A quantitative analysis of mean fluorescence intensity revealed that the cellular uptake of Q-ICG-NLs was at least 17% higher (*p* < 0.01) than that of free ICG at an equivalent concentration of 30 µM ICG (Figure 5B). Since Q-ICG-NLs exhibit negligible fluorescence in their native state (Figure 3B), the comparable fluorescence observed in cells treated with Q-ICG-NLs and free ICG suggests that Q-ICG-NLs were efficiently taken up by the cancer cells. Subsequently, the release of aggregated ICG molecules from the nanoliposomes led to the dequenching of NIR fluorescence of Q-ICG-NLs within the cells.

Clathrin-mediated endocytosis is well known as a major pathway for liposomal internalization [22,23]. When Q-ICG-NL-treated cells were co-stained with LysoTracker, a fluorescent probe for lysosome staining (Figure S3), the fluorescence of internalized Q-ICG-NLs overlapped with that of LysoTracker, indicating that Q-ICG-NLs are internalized via clathrin-mediated endocytosis. Therefore, it appears that the release of aggregated ICG molecules from the nanoliposomes occurs through simple diffusion within lysosomal compartments.

As mentioned above, intravenously injected Q-ICG-NLs are expected to selectively accumulate in tumors via the enhanced permeability and retention (EPR) effect. However, a portion of the injected Q-ICG-NLs may also distribute to normal organs such as the lungs, liver, kidneys, and spleen. To assess their selectivity, we evaluated the cellular uptake of Q-ICG-NLs in the primary renal cortical epithelial cells and compared it with uptake in Calu-3 cells. Confocal fluorescence imaging revealed that Q-ICG-NLs exhibited a 7.2-fold higher uptake in Calu-3 cells compared to the tested normal cells (Figure S4).

### 2.4. In Vitro Dark Toxicity and Phototoxicity Testing

**Figure 6:**
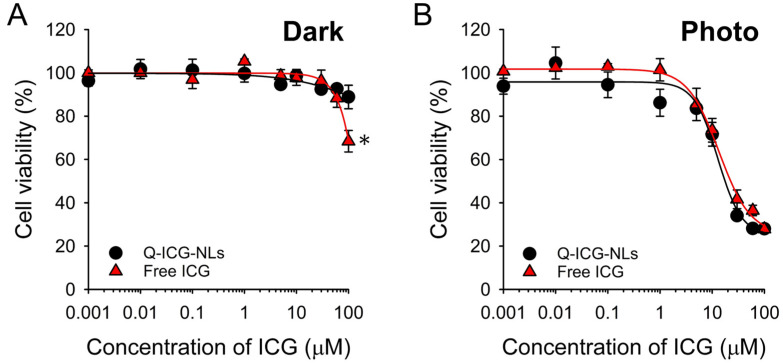
(**A**) Cell viability of Calu-3 cells treated with free ICG or Q-ICG-NLs at various ICG-equivalent concentrations (*n* = 4). (**B**) In vitro phototoxicity test (*n* = 4). Calu-3 cells were treated with free ICG or Q-ICG-NLs for 16 h at various ICG-equivalent concentrations, washed twice, and then irradiated with an 808 nm CW laser (0.5 W/cm^2^ for 10 min). After 24 h, cell viability was measured. * *p* < 0.01.

Before assessing the phototoxicity of Q-ICG-NLs, we first examined their dark toxicity alongside that of free ICG (Figure 6A). Calu-3 cells were treated with free ICG or Q-ICG-NLs at concentrations ranging from 0 to 100 µM. Both treatments exhibited minimal or negligible cytotoxicity at concentrations up to 60 µM. Cells treated with Q-ICG-NLs at 100 µM showed 88% viability, whereas those treated with free ICG at the same concentration exhibited a lower viability of 68% compared to Q-ICG-NL-treated cells (*p* < 0.05).

Next, we evaluated the potential of Q-ICG-NLs for activatable photodynamic therapy (PDT) (Figure 6B). Upon irradiation with an 808 nm continuous-wave (CW) laser, cancer cells treated with Q-ICG-NLs exhibited a dose-dependent therapeutic effect. The IC_50_ of the Q-ICG-NL-treated group was 12.9 µM, which is similar to that of free ICG with an IC_50_ of 13.5 µM. These findings suggest that, despite the larger size of the nanoliposome formulation compared to free ICG, Q-ICG-NLs effectively facilitated cellular uptake and enabled the restoration of the quenched phototoxicity of ICG within the cellular environment.

These findings demonstrate the selective therapeutic efficacy of Q-ICG-NLs against cancer cells. The enhanced therapeutic outcome is likely due to the ability of nanoliposomes to efficiently deliver ICG molecules to cancer cells and restore the phototoxicity of ICG within the cellular environment. Moreover, in vivo, the extended circulation time and improved stability of Q-ICG-NLs compared to free ICG are expected to further enhance their therapeutic effectiveness. As mentioned above, intravenously administered ICG has significant drawbacks as a theranostic agent for cancer, including a short blood half-life (2–4 min), instability in an aqueous environment, and a lack of target specificity [12,13,14,15]. In contrast, Q-ICG-NLs are expected to accumulate in tumors via the enhanced permeability and retention (EPR) effect [24]. Following uptake into cancer cells, the quenched NIR fluorescence and phototoxicity of Q-ICG-NLs could be restored inside cancer cells, enabling tumor-selective generation of NIR fluorescence and image-guided precision phototherapy.

## 3. Materials and Methods

### 3.1. Materials

L-α-phosphatidylcholine, hydrogenated (Soy) (HSPC), and 1,2-distearoyl-sn-glycero-3-phosphoethanolamine-N-[methoxy(polyethylene glycol)-2000] (DSPE-PEG2000-PE) were obtained from Avanti Polar Lipids (Alabaster, AL, USA). Dimethyl sulfoxide (DMSO) and cholesterol and were purchased from Merck Korea (Seoul, Republic of Korea). Indocyanine green (ICG) was acquired from Selleck Chemicals LLC (Houston, TX, USA). An Amicon centrifugal filter unit (molecular weight cut-off 100 kDa, UFC805024) was obtained from Merck Millipore (Darmstadt, Germany). A Sephadex G25 gel filtration column (PD-10) was purchased from Cytiva (Marlborough, MA, USA). Singlet oxygen sensor green (SOSG) was acquired from Invitrogen (Grand Island, NY, USA).

### 3.2. Preparation of Quenched ICG-Loaded Nanoliposomes (Q-ICG-NLs)

First, HSPC, DSPE-PEG2000-PE, and cholesterol were each dissolved in chloroform at a concentration of 10 mg/mL and combined in a round-bottom flask at a molar ratio of 85:10:5, resulting in a total volume of 480 µL. Separately, ICG was dissolved in ethanol at a concentration of 2 mg/mL, and the ICG solution was then added to the lipid mixture. The solution was homogenized using a rotary evaporator, which simultaneously removed the solvent to form a thin lipid film on the inner surface of the flask. Subsequently, 2 mL of deionized water preheated to 65 °C was added to hydrate the lipid film, followed by sonication for 2 min (Brasonic^®^ 2800, Branson, MO, USA). The resulting dispersion was transferred to a glass vial and subjected to further sonication using a probe-type sonicator (Sonicator 3000, Misonix, NY, USA, 1.5 W, 2 s pulse on-off time) for 20 min to ensure uniform nanoliposome formation. To remove unencapsulated ICG and other impurities, the solution was purified using a PD-10 size-exclusion column. Finally, the purified nanoliposome solution was concentrated by centrifugation at 3000 rpm for 20 min at 25 °C using an Amicon^®^ Ultra Centrifugal Filter tube (Merck, NJ, USA).

### 3.3. Characterization of Q-ICG-NLs

The hydrodynamic size of Q-ICG-NLs was measured using a Malvern Zetasizer (Malvern Panalytical, Worcestershire, UK). The concentration of ICG encapsulated in the Q-ICG-NLs was analyzed by dissolving them in DMSO solvent and measuring the absorbance at 820 nm using a UV/Vis spectrophotometer (DU730, Beckman Coulter, Brea, CA, USA). The encapsulation efficiency (EE) of ICG in the Q-ICG-NLs was calculated using the following equation:

$$ICG\;EE\left(\%\right)=\frac{Concentration\;of\;Encapsulated\;ICG}{Concentration\;of\;Initial\;ICG}\times 100\%$$

The UV/Vis absorption spectra of Q-ICG-NLs and free ICG were obtained by dissolving them in deionized water at 30 μM ICG equivalent. The fluorescence spectra of Q-ICG-NLs and free ICG in deionized water (λ_ex._ = 720 nm, λ_em._ = 760–900 nm) were also measured using a multi-mode microplate reader (TECAN SPARK, TECAN, Männedorf, Switzerland). The morphology of Q-ICG-NLs was observed using cryo-transmission electron microscopy (TEM, JEOL JEM-2100Plus, Sollentuna, Sweden).

### 3.4. Measurement of Singlet Oxygen Generation (SOG) During Laser Irradiation

To evaluate SOG, singlet oxygen sensor green (SOSG) was used as a singlet oxygen-detecting reagent. The PBS solution used in this experiment was pre-saturated with oxygen gas, and SOSG was dissolved in the PBS solution at a concentration of 1 μM. ICG samples (free ICG or Q-ICG-NLs at 30 μM ICG equivalent) were prepared using the PBS containing SOSG and then irradiated with an 808 nm CW laser (0.5 W/cm^2^). During light irradiation, the fluorescence intensity of SOSG in the sample solutions was measured every 30 s using a multi-mode microplate reader. For comparison, PBS without ICG was also irradiated with the 808 nm laser, and changes in SOSG fluorescence were measured. All experiments were conducted in quadruplicate.

### 3.5. Measurement of Photothermal Effect During Laser Irradiation

To evaluate the photothermal effect, an experimental setup was established in which an NIR laser was irradiated from the side and thermal imaging was performed from above (Figure 3A). PBS solutions containing free ICG and Q-ICG-NLs were prepared at concentrations of 0, 5, 10, and 30 μM. The sample solutions were added to disposable cuvettes and irradiated with an 808 nm CW laser at 0.5 W/cm^2^. Thermal images were captured every 1 min using a thermographic camera (T440, FLIR, Wilsonville, OR, USA) for 15 min to analyze the temperature changes during laser irradiation.

### 3.6. Cell Culture

The Calu-3 human lung carcinoma cell line was obtained from the American Type Culture Collection (ATCC, Rockville, MD, USA). This cell line was cultured in a growth medium consisting of Eagle’s Minimum Essential Medium (WELGENE), 10% Fetal bovine serum (FBS, Gibco) and 1% Antibiotic-Antimycotic (Thermo Fisher Scientific, Waltham, MA, USA) at 37 °C in a 5% CO_2_ atmosphere.

### 3.7. Confocal Fluorescence Microscopy for Intracellular Uptake

Calu-3 cells were seeded and incubated in LabTek II Chambered Coverglass (Nalge Nunc International Corp., Rochester, NY, USA) at a cell density of 3 × 10^4^ cells/well. The cell culture medium was then replaced with fresh medium containing Q-ICG-NLs or free ICG at a concentration of 30 μM ICG equivalent. After 16 h of treatment, the cells were washed twice with PBS, and fresh culture medium was added. NIR fluorescence images of the cells (λ_ex._ = 633 nm, λ_em._ = 700 ± 50 nm) were acquired using a confocal laser scanning microscope (LSM 880; Carl Zeiss, Oberkochen, Germany).

### 3.8. In Vitro Dark Toxicity and Phototoxicity Testing

Calu-3 cells were seeded and incubated in a 96-well plate at a cell density of 1 × 10^4^ cells/well. Q-ICG-NLs and free ICG were dissolved in cell culture medium at various concentrations (from 0.001 μM to 100 μM ICG equivalent) for dark toxicity testing. The culture medium was then replaced with 100 μL of fresh medium containing Q-ICG-NLs or free ICG (*n* = 4). After 16 h of treatment, the cells were washed twice, and fresh cell culture medium was added. The cells were further incubated for 24 h, and their viability was measured using a CellTiter-Glo assay (Promega Corp., Madison, WI, USA). The absorbance of each sample at 450 nm was recorded using a BioTek Synergy HTX microplate reader (Agilent, Santa Clara, CA, USA). The absorbance values were then converted to relative cell viability (%) by normalizing them to the untreated control cells, which were used as a reference for 100% viable cells.

For phototoxicity testing, Calu-3 cells were seeded and incubated in a 96-well plate at a cell density of 1 × 10^4^ cells/well. Q-ICG-NLs and free ICG were dissolved in cell culture medium at various concentrations (from 0.001 μM to 100 μM ICG equivalent). The culture medium was then replaced with 100 μL of fresh medium containing Q-ICG-NLs or free ICG (*n* = 4). After 16 h of treatment, the cells were washed twice, fresh cell culture medium was added, and the cells were irradiated using an 808 nm CW laser (0.5 W/cm^2^). The cells were further incubated for 24 h, and their viability was measured using a CellTiter-Glo assay. The absorbance of each sample was recorded using a microplate reader.

### 3.9. Statisitcal Analysis

Differences between the groups were evaluated using Student’s *t*-test. Data are expressed as the mean ± standard deviation.

## 4. Conclusions

In this study, we developed quenched ICG-loaded nanoliposomes (Q-ICG-NLs) as activatable theranostic agents and evaluated their potential applications. The Q-ICG-NLs exhibited excellent biocompatibility, a large cavity for drug loading, ease of large-scale synthesis, and high monodispersity in both size and morphology, confirming their effectiveness as drug delivery nanocarriers. In their native state, the NIR fluorescence and singlet oxygen generation of Q-ICG-NLs were significantly quenched, but were largely restored upon uptake into cancer cells, enabling activatable fluorescence imaging and phototherapy. Overall, the Q-ICG-NLs developed in this study hold great promise for the selective NIR fluorescence imaging of cancer and image-guided precision phototherapy.

## Data Availability

The datasets generated and/or analyzed during the current study are available from the corresponding author upon reasonable request.

## Supplementary Materials

The following supporting information can be downloaded at: https://www.mdpi.com/article/10.3390/molecules30071414/s1, Figure S1: Characterization of fluorescence emission of ICG-loaded nanoliposomes prepared at various lipid:ICG molar ratios.; Figure S2: Comparison of the optical properties of Q-ICG-NLs in the absence and presence of SDS surfactant. Figure S3: Evaluation of the cellular uptake pathway of Q-ICG-NLs. Figure S4: Comparison of Q-ICG-NLs uptake between normal and cancer cells.
