# Supplementary material for: Indocyanine Green-Loaded Quenched Nanoliposomes as Activatable Theranostics for Cancer
Source: Molecules. 2025 Mar 22;30(7):1414. doi: 10.3390/molecules30071414 (PMC11990451; doi:10.3390/molecules30071414)
Supplement: Supplementary file 1 [file molecules-30-01414-s001.zip › molecules-3537238-supplementary.pdf]

Supplementary Materials

Indocyanine Green-loaded Quenched Nanoliposomes as Activatable Theranostics for Cancer

Junwoo Lim, Yeojin Yoo, Yongdoo Choi,*

Division of Technology Convergence, National Cancer Center, 323 Ilsan-ro, Goyang, Gyeonggi-Do 10408, Republic of Korea; jwlim@ncc.re.kr (J.L.); yooyeos@ncc.re.kr (Y.Y.)

* Correspondence: ydchoi@ncc.re.kr (Y.C.)

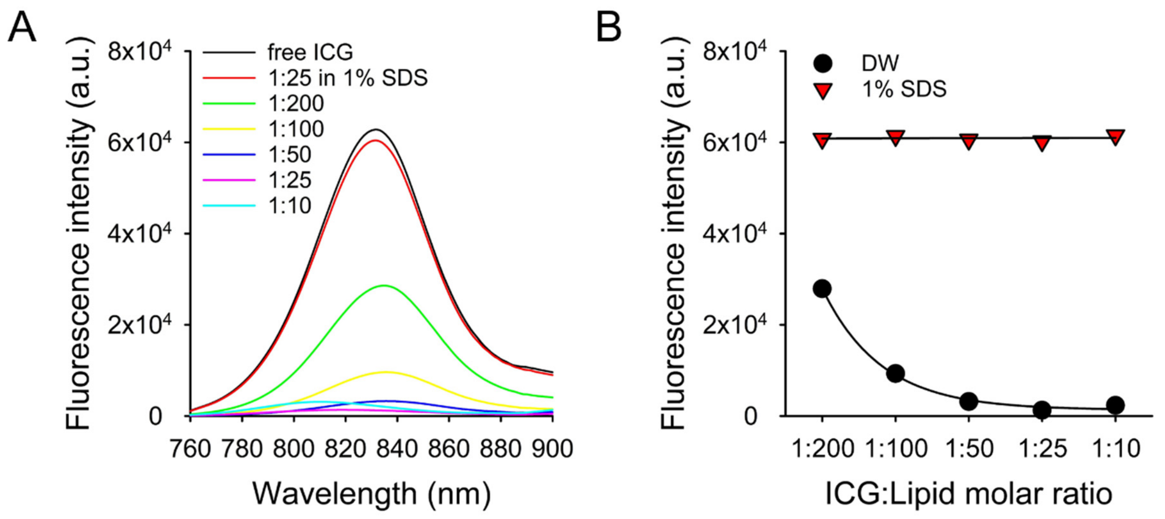

Figure S1. Characterization of fluorescence emission of ICG-loaded nanoliposomes prepared at various lipid:ICG molar ratios. (A) Fluorescence spectra ($\lambda_{ex.}$=720 nm) of free ICG and ICG-loaded nanoliposomes at different ICG:lipid molar ratios (ranging from 1:10 to 1:200). (B) Comparison of fluorescence intensity ($\lambda_{ex.}$=720 nm, $\lambda_{em.}$=830 nm) of ICG-loaded nanoliposomes in deionized water (DW) and DW containing 1%(w/v) SDS. The nanoliposome prepared with an ICG-to-lipid molar ratio of 1:25 exhibited the highest quenching effect, showing a 52.9-fold difference.

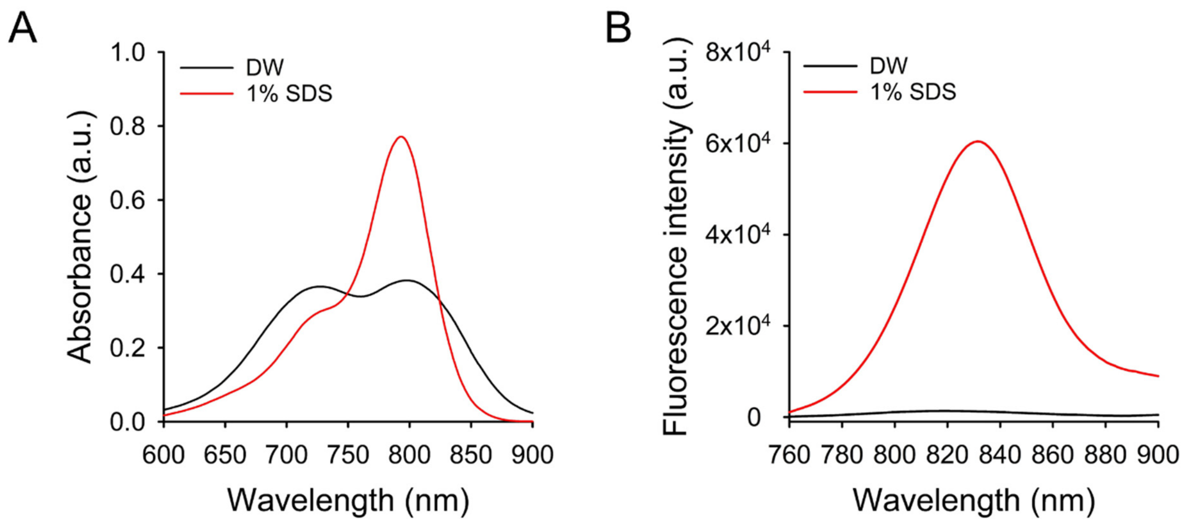

Figure S2. Comparison of the optical properties of Q-ICG-NLs in the absence and presence of SDS surfactant. (A) Absorption and (B) fluorescence spectra of Q-ICG-NLs in DW and DW containing 1%(w/v) SDS.

Confocal fluorescence imaging for evaluation of the cellular uptake pathway

To investigate the cellular uptake pathway of Q-ICG-NLs in Calu-3 cells, lysosomes were co-stained with LysoTracker™ Blue DND-22 (Invitrogen™, Waltham, U.S.) and analyzed. The experiment was conducted as follows: Calu-3 cells were seeded in LabTek II Chambered Coverglass (Nalge Nunc International Corp., USA) at a density of **3 × 10⁴** cells per well and incubated. The cell culture medium was then replaced with fresh medium containing Q-ICG-NLs at a concentration of 30 μM ICG equivalent. After 4 hours of treatment, the cells were washed twice with PBS, and lysosomes were stained with LysoTracker™ Blue.

Near-infrared (NIR) fluorescence images of the cells (ICG: $\lambda_{ex.}$ = 633 nm, $\lambda_{em.}$ = 700 ± 50 nm; Lysotracker: λ_{ex} = 374 nm, $\lambda_{em.}$ = 422 nm) were acquired using a confocal laser scanning microscope. The fluorescence intensity profile along the arrow direction in **Figure S3E** demonstrates overlapping intensity patterns of ICG and LysoTracker, indicating their co-localization.

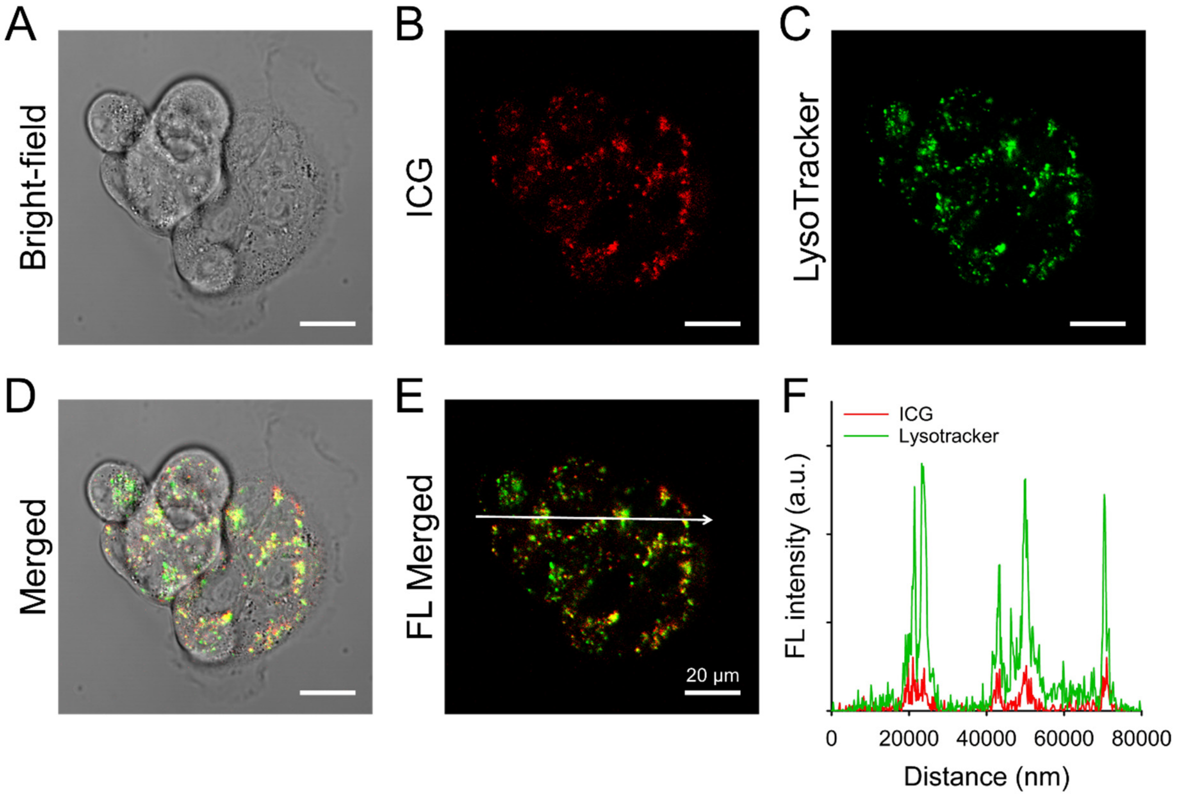

Figure S3. Evaluation of the cellular uptake pathway of Q-ICG-NLs. (A–E) Confocal fluorescence images of Calu-3 cells treated with Q-ICG-NLs and LysoTracker. The yellow-colored region in Figures S3D and S3E indicates the overlap of ICG fluorescence and LysoTracker staining, suggesting co-localization. (F) Fluorescence intensity profile analysis of Calu-3 cells treated with Q-ICG-NLs and LysoTracker along the arrow indicated in Figure S3E. Scale bar = 20 μm.

Comparison of Q-ICG-NLs uptake between normal and cancer cells

To evaluate the selective therapeutic potential of Q-ICG-NLs for cancer cells, their cellular uptake was analyzed and compared between normal and cancer cells. The experiment was conducted as follows: Primary human renal cortical epithelial (HRCE) cells, a normal renal cortical epithelial cell line, were obtained from the American Type Culture Collection (ATCC, Rockville, MD, USA). These normal cells were cultured in Renal Epithelial Cell Basal Medium supplemented with the Renal Epithelial Cell Growth Kit (both from ATCC) at 37°C in a 5% CO_2 atmosphere. Calu-3 cells and HRCE cells were seeded in LabTek II Chambered Coverglass (Nalge Nunc International Corp., USA) at a density of 3×10^4 cells per well and incubated. The cell culture medium was then replaced with fresh medium containing Q-ICG-NLs at a concentration of 30 μM ICG equivalent. After 16 hours of treatment, the cells were washed twice with PBS, and fresh culture medium was added.

Near-infrared (NIR) fluorescence images ($\lambda_{ex.}$ = 633 nm, $\lambda_{em.}$ = 700 ± 50 nm) were acquired using a confocal laser scanning microscope. As shown in Figure S4A, the fluorescence intensity of ICG was significantly higher in Calu-3 cells compared to HRCE cells. Quantitative fluorescence analysis revealed that the cellular uptake of Q-ICG-NLs in Calu-3 cells was 7.2-fold higher than in HRCE cells (Figure S4B).

These findings indicate that Q-ICG-NLs are more efficiently internalized by Calu-3 cells, enabling selective cancer treatment. Furthermore, the lower cellular uptake in HRCE cells suggests a significant reduction in phototoxicity and potential side effects in normal tissues.

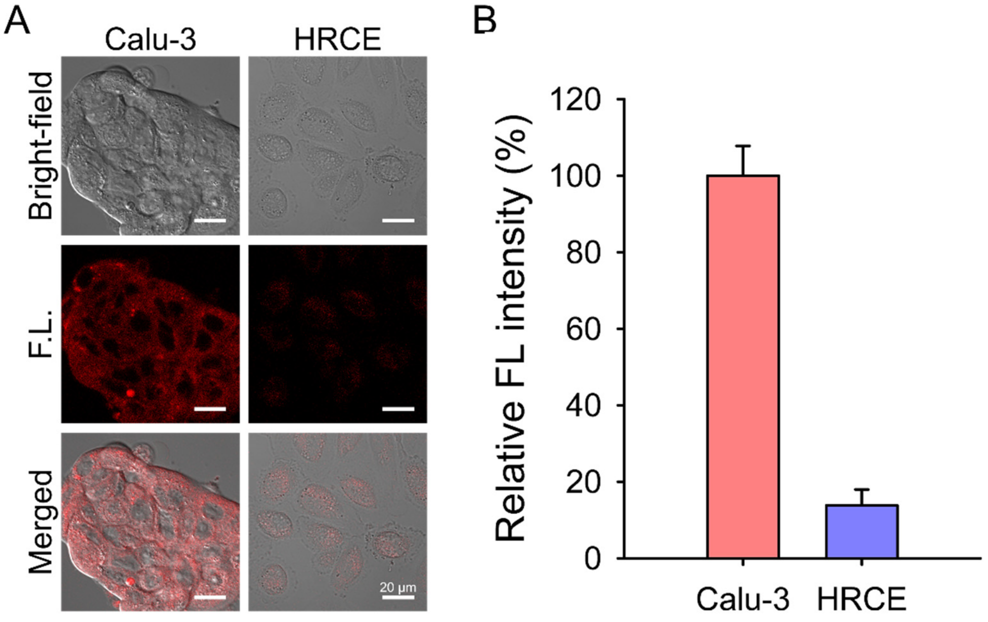

Figure S4. Comparison of Q-ICG-NLs uptake between normal and cancer cells. (A) Confocal fluorescence images of Calu-3 and HRCE cells treated with Q-ICG-NLs ($\lambda_{ex.}$ = 633 nm, $\lambda_{em.}$ = 700 ± 50 nm). (B) Relative fluorescence intensity of near-infrared (NIR) fluorescence signals in Calu-3 and HRCE cells treated with Q-ICG-NLs. Scale bar = 20 μm.
